# Nrf2 drives oxidative stress-induced autophagy in nucleus pulposus cells via a Keap1/Nrf2/p62 feedback loop to protect intervertebral disc from degeneration

**DOI:** 10.1038/s41419-019-1701-3

**Published:** 2019-07-01

**Authors:** Zehan Tang, Bo Hu, Fazhi Zang, Jianxi Wang, Xingda Zhang, Huajiang Chen

**Affiliations:** Department of Spinal Surgery, Changzheng Hospital, Second Military Medical University, Shanghai, China

**Keywords:** Autophagy, Cell signalling

## Abstract

Intervertebral disc (IVD) degeneration is known to aggravate with age and oxidative stress is implicated in the pathogenesis of many age-related diseases. Nuclear factor (erythroid-derived-2)-like 2 (Nrf2) can confer adaptive protection against oxidative and proteotoxic stress in cells. In this study, we assessed whether Nrf2 can protect against oxidative stress in nucleus pulposus (NP) cells. In addition, we investigated Nrf2 expression in NP tissue samples from patients with different degrees of IVD degeneration and a mouse model of aging and IVD degeneration and the influence of H_2_O_2_-induced oxidative stress on autophagic pathways in NP cells. Autophagy was assessed by measuring levels of autophagy-related protein (ATG) family members and the autophagic markers, p62 and LC3. We found that expression of Nrf2 progressively decreased in human NP tissue samples of patients with increasing degrees of IVD degeneration. Nrf2 deficiency leads to the degeneration of IVDs during aging. Nrf2 knockout also aggravates IVD degeneration and reduces autophagic gene expression in an induced mouse model of IVD degeneration. The detrimental effects of H_2_O_2_-induced oxidative stress were increased in autophagy-deficient cells via reduced expression of Atg7 and the Keap1–Nrf2–p62 autophagy pathway. Taken together, these results suggest that excessive oxidative stress causes the upregulation of autophagy, and autophagy acts as an antioxidant feedback response activated by a Keap1-Nrf2-p62 feedback loop in IVD degeneration.

## Background

Intervertebral discs (IVDs) are subject to degeneration as they age, owing to changes in the abundance and structure of macromolecules^1,2^. The center of the disc contains the nucleus pulposus (NP), which is a highly hydrated gelatinous aggrecan-rich core (Urban and Roberts^3^). The NP is surrounded by collagen I-rich fibrous cartilage known as the annulus fibrosus (AF). With increasing age, the NP becomes more fibrous and less gelatinous and the boundary between the NP and AF appears less distinct^4^. In addition, the disc develops a disorganized morphology combined with an accumulation of cell waste products and degraded matrix molecules^3,4^. The inability of the mature disc to remove or replace accumulated degradation products is believed to contribute to IVD degeneration^1^.

Several pathways are involved in the degradation and maintenance of proteins, including endocytosis, crinophagy, macroautophagy, microautophagy, and chaperone-mediated autophagy^5^. Autophagy is an evolutionarily conserved homeostatic process involved in quality control and the recycling of cell components^6^. The autophagic process is initiated by a complex involving unc-51-like kinase 1 (ULK1) and members of the autophagy-related protein (ATG) family^7^. The ULK1 complex translocates to autophagy initiation sites where it recruits the involvement of the vacuolar protein sorting 34 (VPS34) complex, which includes BECLIN-1, VPS15, and ATG14L^7,8^. The VPS34 complex proteins are involved in the formation of the phagophore, which is further processed into an autophagosome by another complex, believed to include ATG16L1, ATG5, and ATG12^9^.

Genes that are associated with autophagy are known to be upregulated in human degenerated IVDs^6^. However, it is not clear whether the upregulation of autophagy is associated with cell death or survival. Autophagy is mainly considered a process was organelles and cytosolic proteins, encapsulated within autophagosomes, are degraded by lysosomes. In cell survival, autophagy-mediated degradation can be rapidly activated and provide the amino acids to support metabolism and macromolecular synthesis and remove toxic protein aggregates from the cell (10.1016/j.molcel.2014.07.019 Mathew et al. 2014). The activation of autophagy in rats is thought to ameliorate the progression of osteoarthritis^10^.

In addition to aging, IVDs are prone to various stresses^11^. The nutrient supply of the NP and exportation of metabolic wastes depends on diffusion through the cartilaginous endplate and AF, which can become disrupted by mechanical stress and calcification^12^. Therefore, NPs, in particular, are challenged with nutrient deprivation, acidic stress, hyperglycemia, hypoxia, reactive oxygen species (ROS), and proinflammatory cytokines^11^. These stresses not only affect proliferation, growth and protein synthesis but can also induce autophagy^13^. The autophagic pathways that are most associated with age are macroautophagy and chaperone-mediated autophagy^14^. Levels of macroautophagy and chaperone-mediated autophagy were found to be elevated in rat NPs with increasing age^15^.

Oxidative stress is associated with the pathogenesis of many age-related diseases, including IVD degeneration^16^. In human NP cells, oxidative stress inhibits proliferation, induces premature senescence, and promotes a catabolic phenotype^17^. Nuclear factor (erythroid-derived-2)-like 2 (Nrf2), is known to confer adaptive protection against oxidative and proteotoxic stress in cells^18^. The induction of NRF2 genes requires a common NRF2-binding motif known as the antioxidant response element (ARE)^19^. Nrf2 is believed to maintain cellular homeostasis when cells are exposed to reactive oxygen or nitrogen species generated by metabolism or stress. A recent study suggests that Nrf2 is involved in the fine-tuning of the autophagic process in response to the level of oxidative stress and functions in a feedback loop in combination with AMP-activated protein kinase (AMPK), which is crucial for autophagy induction via mTOR down-regulation^20^. In auditory cells under oxidative stress, an autophagic pathway was found to be maintained by a Kelch-like ECH-associated protein 1 (Keap1)–Nrf2 feedback loop through p62, a protein encoded by the sequestosome 1 gene (*SQSTM1*)^21^. Moreover, in a study assessing the anti-cancer properties of the drug isodeoxyelephantopin (ESI), p62 induced by ESI was found to bind competitively with Keap1 to release Nrf2^22^. Nrf2 then activates downstream target genes, including p62, to promote autophagy in a positive feedback loop. Under normal conditions, Keap1 is bound to Nrf2 and constantly degraded through an ubiquitin–proteasome pathway^23^. In response to oxidative stress, Nrf2 is released from the Nrf2–Keap1 complex, through p62 binding to Keap1 competitively, and then translocates to the nucleus, where it activates the transcription of downstream target genes^23^. The p62 protein is constantly degraded by autophagy, therefore, elevated p62 levels in the absence of increased *SQSTM1*expression, indicates dysfunctional autophagy^24^. Microtubule-associated protein 1A/1B-light chain 3 (LC3), is also used as an autophagic marker^25^. During autophagy, LC3-I is recruited from the cytosol to the autophagosomal membrane to form the LC3-phosphatidylethanolamine conjugate (LC3-II); p62/SQSTM1 acts as a link between LC3 and ubiquitinated substrates^26^.

In this study, we assess whether Nrf2 can confer an adaptive protection against oxidative and proteotoxic stress in NP cells. In addition, Nrf2 knockout was assessed in a mouse model of IVD degeneration and the influence of H_2_O_2_-induced oxidative stress on the Keap1-Nrf2-p62 pathway and autophagy was also investigated in autophagy-deficient cells.

## Results

### Decreased expression of Nrf2 in human NP cells in IVD degeneration

**Fig. 1:**
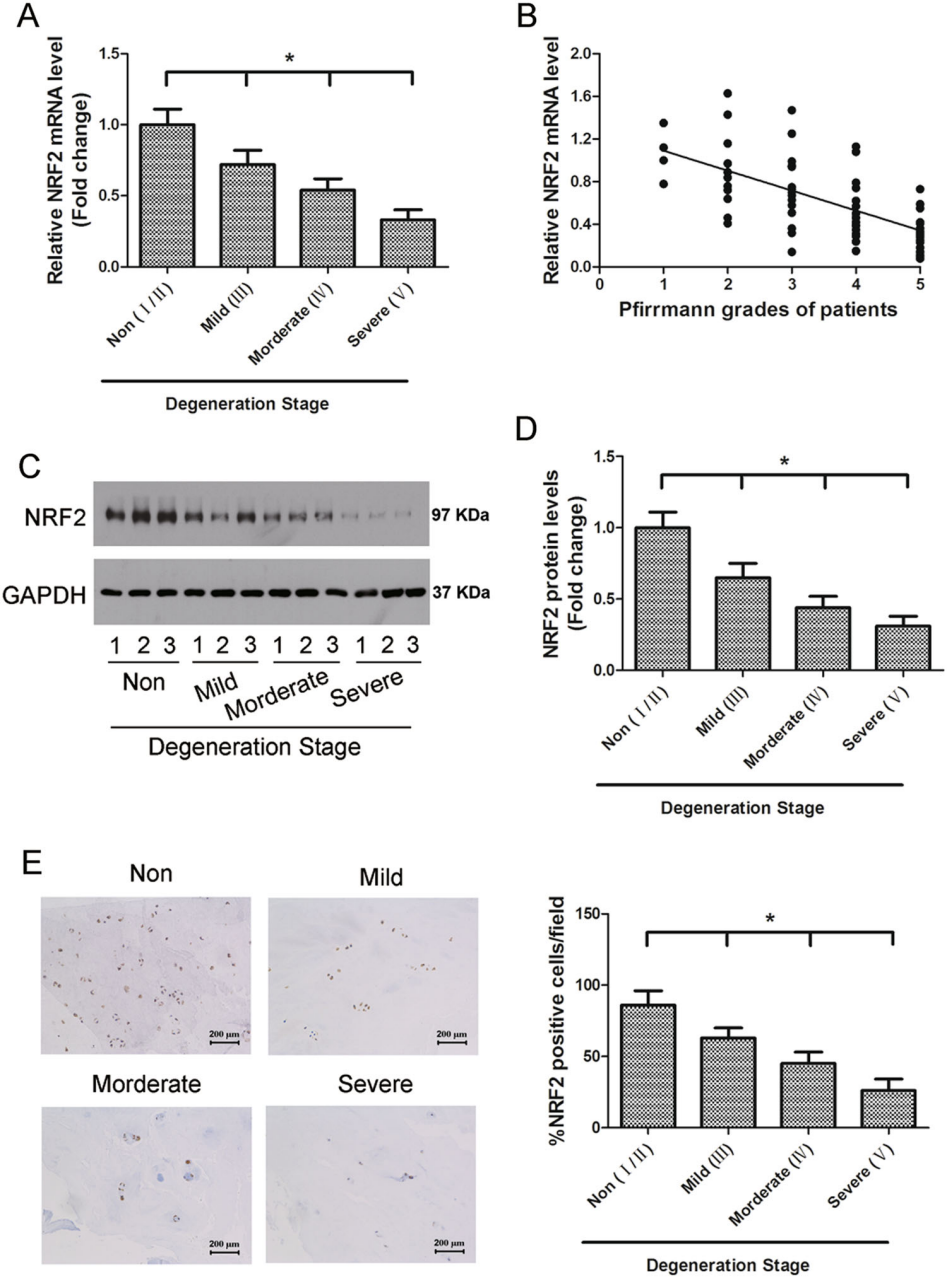
The expression of Nrf2 in human nucleus pulposus (NP) tissues. **a** Real-time RT-PCR analysis showed expression level of Nrf2 mRNA decreased with the severity of IVD degeneration. **b** Gene expression of Nrf2 was negatively correlated with the Pfrrmann grades of 48 human NP tissues (*n* = 60, *r* = −0.623). **c** Representative western blot of Nrf2 in human NP tissue (*n* = 12, 3 for each grade of IVD). **d** Densitometry analysis of at least three western blot experiments shown in (**c**). **e** Immunohistochemistry of the human NP sections showed that the numbers of Nrf2-positive cells were decreased with IVD degeneration. Grade 2 represents non-degenerated IVD, whereas grades 3, 4, and 5 signify mild, moderate, and severe degeneration, respectively. Values are shown as the mean and standard deviation (SD). *P < 0.05, bars = 100 μm

NP tissue samples from patients with different degrees of IVD degeneration were divided into four groups according to a previously described classification system^27^: non-degenerated (grade I/II, *n* = 15), mildly degenerated (grade III, *n* = 15), moderately degenerated (grade IV, *n* = 15), and severely degenerated (grade V, *n* = 15) (Fig. 1). The relative mRNA expression of Nrf2 was found to be negatively correlated with the Pfrrmann grades of NP tissues (*n* = 60, *r* = −0.623), patients with grade V disc degeneration presented the lowest expression of Nrf2 (Fig. 1a, b). Western blotting confirmed these findings, with the lowest level of Nrf2 protein found in grade V tissues (Fig. 1c, d). Moreover, immunohistochemistry demonstrated that there were significantly fewer Nrf2-positive cells in human NP sections with severe IVD degeneration (Fig. 1e, f). These results show that lower levels of Nrf2 correlates with higher severity of IVD degeneration.

### Knockout of Nrf2 leads to degeneration in IVDs during aging

**Fig. 2:**
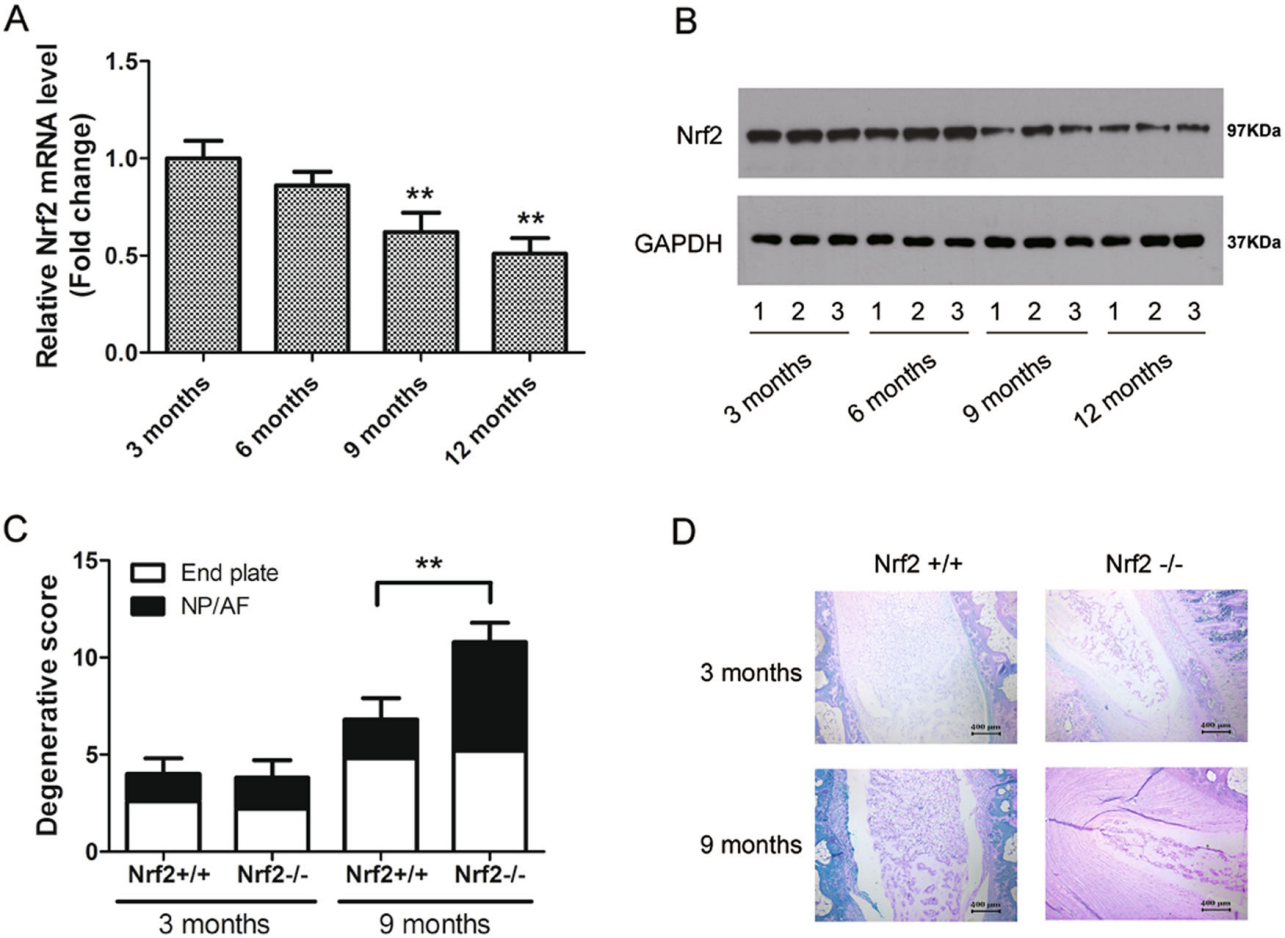
Knockout of Nrf2 leads to the degeneration of invertebral discs (IVDs) during aging. **a** RNA level of Nrf2 in 3, 6, 9, and 12-month-old mice (*n* = 5, respectively), assayed by real-time PCR. The relative unit of Nrf2 expression for 3-month-old mice was set to 1. *P < 0.05. **b** Protein level of Nrf2 in IVD of 3, 6, 9, and 12-month-old mice (*n* = 5, respectively), assayed by western blotting. **c** Histological degenerative scores of IVDs in Nrf2+/+ and Nrf2−/− mice aged 3 months and 9 months. Histological changes were observed by hematoxylin and eosin (HE). **d** Analysis of the proteoglycan extracellular matrix composition by Safranin O staining of sections from the intervertebral discs (IVDs) in Nrf2+/+ and Nrf2−/− mice aged 3 months and 9 months

To further understand the role of Nrf2 in physiological aging, we examined its expression in the IVDs of mice from 3 months to 1 year of age. Real-time RT-PCR and western blot analysis showed that the expression levels of Nrf2 decreased with age in IVDs and were significantly lower from 9 months (Fig. 2a, b). We used a previously described method to score the histological degeneration of NP, AF, and endplates in the IVDs of the mice^28^. The histological degenerative scores of IVDs in Nrf2+/+ and Nrf2−/− mice aged 3 and 9 months are shown in Fig. 2c. The histological score progressed significantly in Nrf2−/− mice at the age of 9 months although no significant difference was found at 3 months. Safranin Ostaining in the Nrf2+/+ and Nrf2−/− mice aged 3 months, identified clusters of cells with slight condensation of proteoglycan-rich extracellular matrix, whereas the 9-month Nrf2−/− mice showed relatively faint NP staining compared to the Nrf2+/+ mice (Fig. 2d), suggesting a decrease in the proteoglycan content.

### Nrf2 knockout aggravates IVD degeneration and reduces autophagy gene expression in a mouse model of induced IVD degeneration

**Fig. 3:**
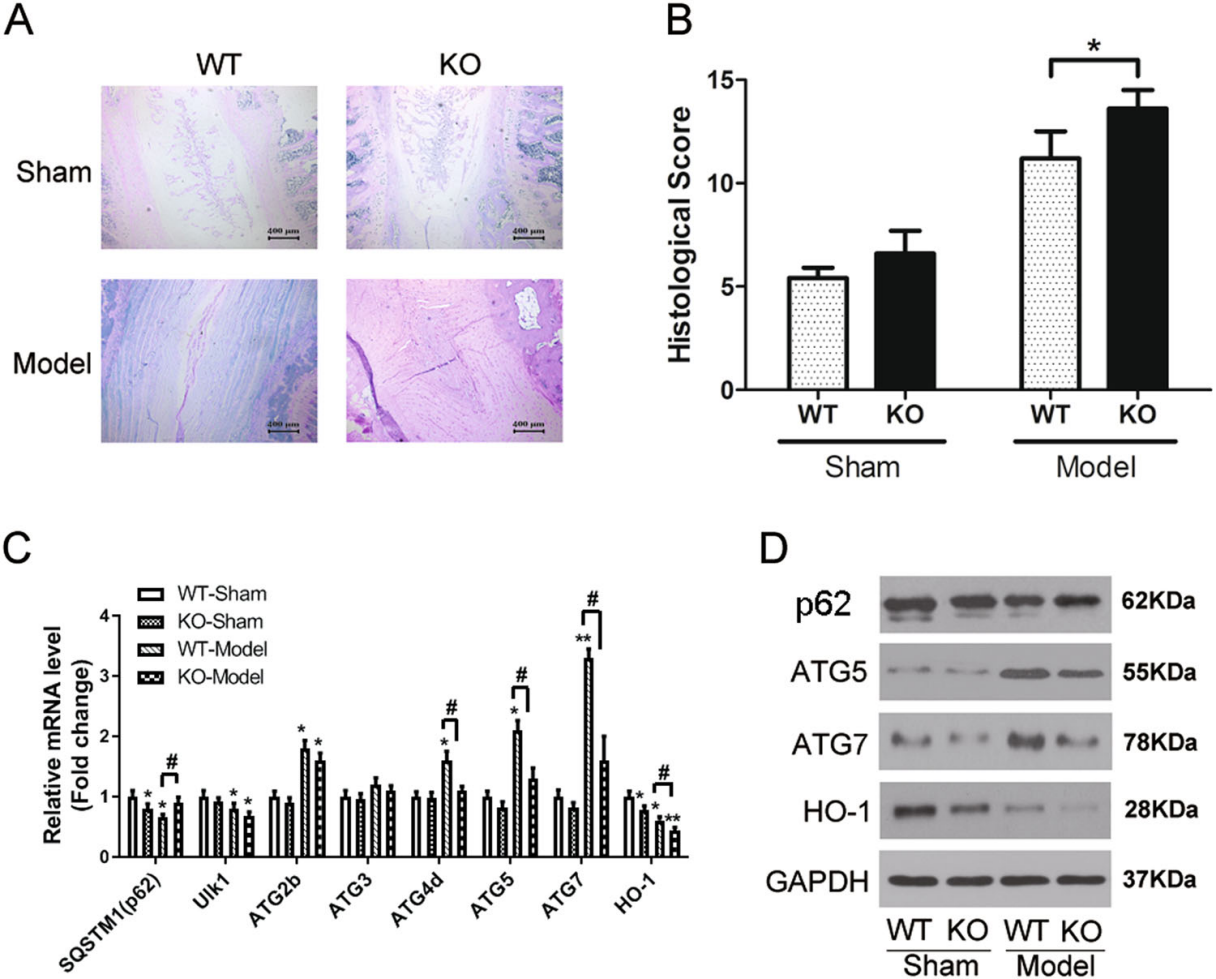
Knockout of Nrf2 aggravates invertebral disc (IVD) degeneration and reduces autophagy genes expression in induced mouse model of IVD degeneration. **a** Analysis of extracellular matrix composition by Safranin O staining of sections from IVDs in WT and Nrf2-KO mice after puncture (Model) or sham-operation. **b** Histological degenerative scores of IVDs in WT and Nrf2-KO mice after puncture (Model) or sham-operation. Histological changes were observed by hematoxylin and eosin (HE). **c** Expression levels of the autophagy genes from the NP tissues in WT and Nrf2-KO mice after puncture (Model) or sham-operation were determined by qRT-PCR. Data are mean ± SD (*n* = 5). Statistical analysis was performed with the Student’s *t* test. *,#*p* < 0.05; **p < 0.01; * vs. WT-Sham, # vs. WT-Model. **d** Western blotting for the protein levels of SQSTM, Atg5, Atg7, and HO-1

To observe the impact of Nrf2 in vivo, we induced IVD degeneration in mice using a puncture method as previously described^29^. Safranin Ostaining of disc tissue revealed that almost no degenerative process can be observed in the discs of sham-operated mice whereas discs of degeneration-induced mice displayed characteristic disorganized tissue (Fig. 3a). There is a lack of distinct boundary between the NP and AF with a decrease in the volume of notochordal cells in the NP and an increase in chondrocytes. Nrf2-KO produced a slight increase in the inward bulging of the disc but there was no significant difference in the histological degenerative scores between Nrf2-KO and sham-operated mice. After puncture-induced degeneration, Nrf2-KO mice have a higher degenerative score than WT mice (Fig. 3b). Expression levels of autophagy genes were determined by qRT-PCR and western blotting in the induced-model, Nrf2-KO, and respective control mice (Fig. 3c, d). The expression of autophagic-related genes, in particular, Atg5 and Atg7, were significantly upregulated in the mice with induced IVD degeneration. However, in the IVD degeneration model with Nrf2-KO, Atg5, and Atg7 were not upregulated. In contrast, HO1 and ULK1 were significantly downregulated in the Nrf2-KO and WT mice with degenerative discs. There was no difference in the expression of **p62** between the sham-operated mice and Nrf2-KO mice with IVD degeneration although p62 was downregulated in the WT mice with IVD degeneration.

Nrf2 is recently reported being involved in the autophagic process and functioning through Keap1–Nrf2–p62 feedback loop positively in response to the level of oxidative stress^20,21^. **p62** was also found to be one of target genes of Nrf2 in autophagy^22^. The p62 protein is constantly degraded by autophagy, therefore, increased p62 levels were found in Nrf2-KO mice compared to WT mice with IVD degeneration. Overall, these results indicate that Nrf2 knockout aggravates disc degeneration and reduces autophagy.

### H_2_O_2_-induced oxidative stress was increased in autophagy-deficient cells via Atg7 and the Keap1–Nrf2–p62 autophagic pathway

**Fig. 4:**
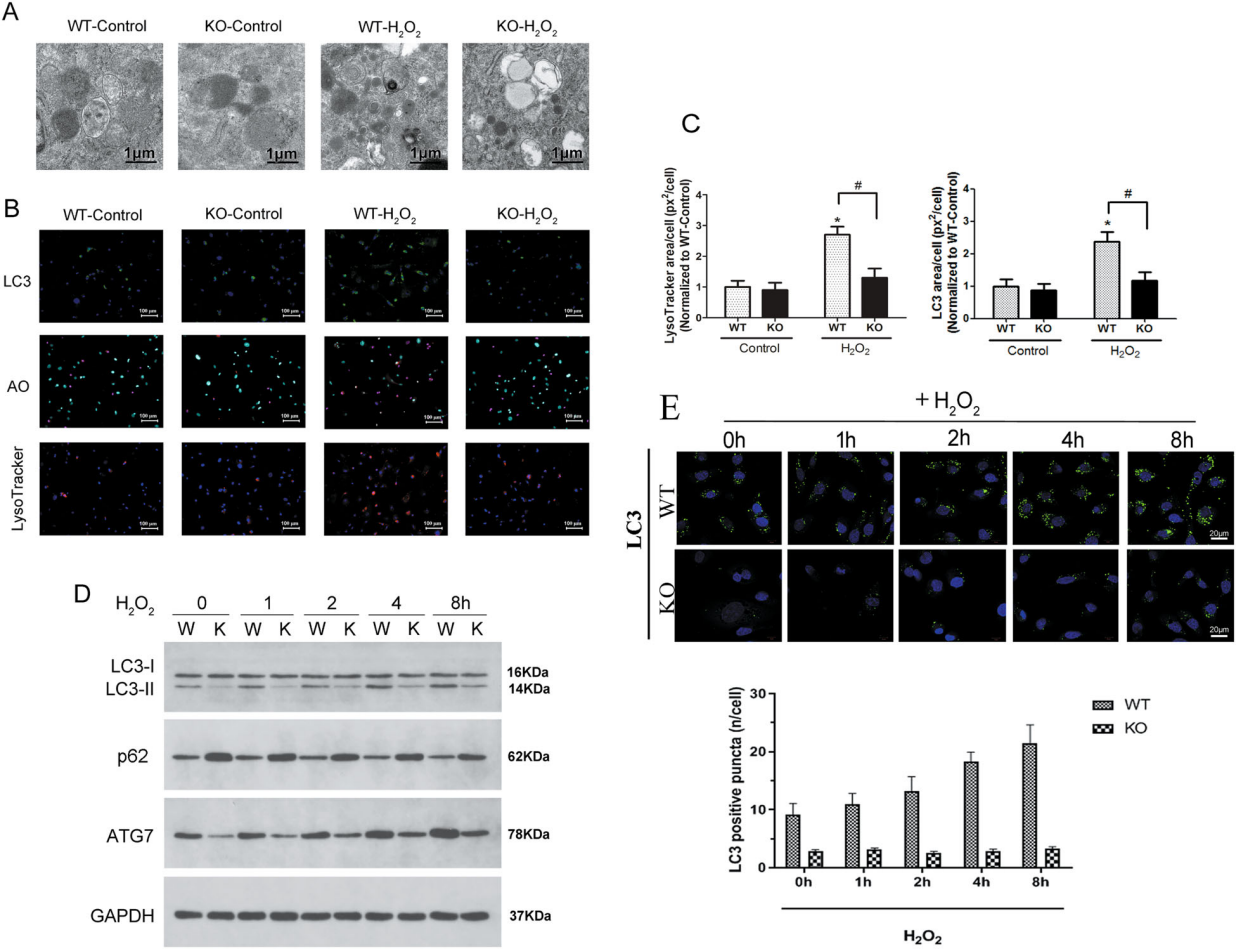
Knockout of Nrf2 increases H_2_O_2_-induced autophagy in nucleus pulposus (NP) cells. **a** Transmission electron microscopy images of primary NP cells treated with or without (control) 400 μM H_2_O_2_ for 2 h show multiple double-membrane enclosed autophagosomes; an increased number are observed in cells with H_2_O_2_ and a decreased number are observed in Nrf2-knockout (KO) cells with H_2_O_2_. Scale bar: 500 nm. **b** Upper panel: Immunofluorescence image of endogenous LC3 in WT or KONP cells indicate increased LC3 puncta under H_2_O_2_-induced oxidative stress. KO of Nrf2 reduced LC3 puncta compared to WT-NP cells under H_2_O_2_-induced oxidative stress. Middle panel: Acridine orange staining of NP cells shows WT cells treated with 400 μM H_2_O_2_ for 2 h have more acidic organelles (red signal) Nrf2-KO reduced acidic organelles under H_2_O_2_-induced oxidative stress. Lower panel: LysoTrackerRed DND-99 staining of NP cells indicates increased red staining after 2 h H_2_O_2_ in WT-NP cells. Nrf2-KO cells have reducedred staining after 2 h H_2_O_2_ culture. **c** Quantification of LysoTracker staining (red) and LC3 (green) as puncta area (px^2^)/cell using ImageJ software confirms increased acidic organelles under H_2_O_2_. At least 427 cells per group were used for quantification analysis. *,#*p* < 0.05; * vs. WT-Control, # vs. WT-H_2_O_2_. **d** Western blotting for the protein levels of LC3-II, SQSTM, and Atg7. **e** LC3 marked autophagosomes were observed by confocal microscopy. WT- and Nrf2-KO-NP cells were incubated in 400 μM H_2_O_2_ for different time periods (0–8 h). The cells without H_2_O_2_ treatment served as a control. *p < 0.05; **p < 0.01; * vs. WT

We further investigated whether Nrf2 may regulate autophagy under oxidative stress. In order to assess the effect of Nrf2 signaling and oxidative stress on autophagy, WT and Nrf2-KO NP cells were incubated in 400 μM H_2_O_2_. Transmission electron microscopy (TEM) images of primary NP cells treated with H_2_O_2_ for 2 h show multiple double-membrane enclosed autophagosomes but a decreased number of autophagosomes were observed in Nrf2-KO cells treated with H_2_O_2_ (Fig. 4a). Moreover, immunofluorescence indicates there are increased LC3 puncta under H_2_O_2_-induced oxidative stress in WT NP cells compared with Nrf2-KO NP cells (Fig. 4b). Acridine orange staining of WT NP cells treated with H_2_O_2_ revealed more acidic organelles compared with Nrf2-KO NP cells under the same conditions. Similar results were obtained with LysoTrackerRed DND-99 staining of NP cells. After WT-NP cells were cultured with H_2_O_2_ the red staining was more intense than that in Nrf2-KO NP cells cultured under the same conditions (Fig. 4b, c). Western blotting indicated reduced protein levels of LC3-II and Atg7 in Nrf2-KO cells whereas **p62** levels were increased compared to WT cells (Fig. 4d). It is worth noting that number of autophagosomes marked by LC3 were increased timed-dependently under H_2_O_2_-induced oxidative stress in WT NP cells and showed no changes in KO NP cells (Fig. 4e). These results indicate that Nrf2 may be required for H_2_O_2_-induced autophagy in NP cells.

**Fig. 5:**
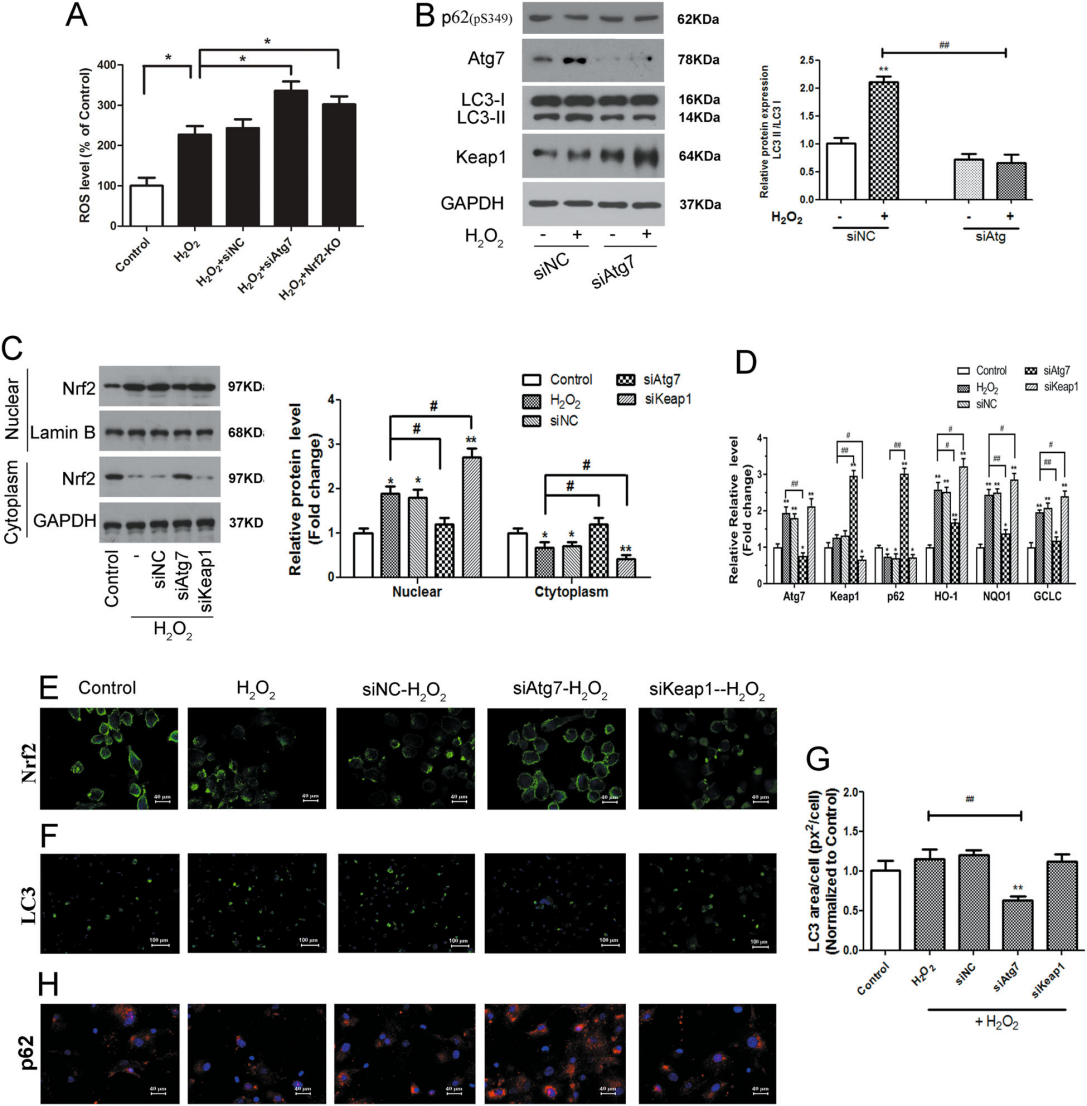
H_2_O_2_-induced oxidative stress was increased in autophagy-deficient cells via Atg7 and Keap1–Nrf2–p62 autophagy pathway. **a** Autophagy-deficient cells have higher levels of ROS. Knockout of Nrf2 and autophagy impaired by Atg7 knockdown increased the elevated ROS level in NP cells stimulated with 400 μM H_2_O_2_ for 2 h. *p < 0.05. **b** NP cells were transfected with Atg7 siRNA or control siRNA 24 h before H_2_O_2_ treatment. Cells were treated with 400 µM of H_2_O_2_ for 2 h. Level of **p62**(pS349), Atg7 and LC3-II and Keap1 degradation was analyzed by western blotting. Atg7 knockdown blocked the accumulation of LC3-II and inhibited Keap1 degradation induced by H_2_O_2_ exposure. ##, **p < 0.01; * vs. siNC- H_2_O_2_(−), # vs. siNC- H_2_O_2_(+). **c** NP cells transfected with Atg7 siRNA, Keap1 siRNA, control siRNA or WT NP cells were treated with H_2_O_2_ (400 μM) for 2 h, and then the nuclear and cytoplasmic fractions were isolated for western blotting. Lamin B and GAPDH were used as markers for nucleus and cytoplasm, respectively. Note that Atg7 siRNA inhibited while Keap1 siRNA promoted the nuclear translocation of Nrf2 induced by H_2_O_2_. *, #*p* < 0.05; **p < 0.01; * vs. Control, # vs. H_2_O_2_ induced cells. **d** NP cells transfected with Atg7 siRNA, Keap1 siRNA, control siRNA or WT NP cells were treated with H_2_O_2_ (400 μM) for 2 h, and the mRNA expression of Atg7, Keap1, **p62**, HO-1, NQO1 and GCLC was measured by qPCR. **e** Confocal microscopy confirmed the nuclear translocation of Nrf2. Green, Nrf2; Blue, DAPI. **f** Fluorescent microscopy for the formation (Green, LC3; Blue, DAPI) and distribution of LC3. *, #*p* < 0.05; ##, **p < 0.01; * vs. Control, # vs. H_2_O_2_ induced cells. **g** LC3 punctate was quantified as LC3 area per cell (px^2^/cell) normal to control. ##, **p < 0.01; * vs. Control, # vs. H_2_O_2_ induced cells. **h** Confocal microscopy confirmed the translocation of **p62** Red, **p62**; Blue, DAPI. Atg7 siRNA reduced while Keap1 siRNA increased LC3 puncta which was contrary to **p62** under H_2_O_2_-induced oxidative stress

To determine whether autophagy can modulate H_2_O_2_-induced oxidative stress, ROS production was measured in autophagy-deficient cells and Nrf2-KO cells. Knockout of Nrf2 and autophagy impaired by Atg7 knockdown increased the elevated ROS level in NP cells stimulated with H_2_O_2_ (Fig. 5a). Atg7 knockout blocked the accumulation of LC3-II, and Nrf2 in nuclear of NP cells induced by H_2_O_2_ exposure (Fig. 5b–e). Depletion of Atg7 inhibits Keap1 degradation (Fig. 5b), and Nrf2 signaling is activated in NP cells under H_2_O_2_-induced oxidative stress, which is confirmed by mRNA expression of Nrf2 target genes as HO-1, NQO1, and GCLC measured by qPCR (Fig. 5c, d). Depletion of Keap1 promotes H_2_O_2_-induced autophagy (Fig. 5d) and increases transcriptional activity of the antioxidant Nrf2 and its target genes as HO-1, NQO1, and GCLC (Fig. 5c, d). Atg7 siRNA reduced while Keap1 siRNA increased LC3 expression (Fig. 5f) which was contrary to **p62** (Fig. 5h) in NP cells under H_2_O_2_-induced oxidative stress. Overall, these results indicate that H_2_O_2_-induced oxidative stress was increased in autophagy-deficient cells via an autophagic pathway modulated by Atg7 and the Keap1–Nrf2–p62.

## Discussion

Degenerative disc disease can lead to a lower quality of life or even disability in severe cases, especially in elderly populations^30^. Therefore, finding solutions to alleviate the symptoms is imperative. It is well known that excessive apoptosis and autophagy are associated with IVD degeneration^6,15^. However, the presence of carboxymethyl-lysine in aging discs has indicated that oxidative stress may also be a contributory factor to disc degeneration^17^. Dimozi et al.^17^ found that subcytotoxic concentrations of H_2_O_2_ could increase ROS intracellular levels, activate stress-associated pathways and induce the nuclear translocation of NF-κΒ and Nrf2^17^.

In this study, we evaluated the expression of Nrf2 in NP tissue samples of patients with degenerated IVDs and found that the severity of the IVD degeneration was negatively correlated with the level of Nrf2, patients with grade V disc degeneration presented the lowest expression of Nrf2. This implies that reduced levels of Nrf2 could contribute to the severity of the disc degeneration. We confirmed that this could be a possibility by investigating the effects of reduced Nrf2 on IVD degeneration in mice. We scored the histological degeneration of the NP, AF, and endplates in mice aged between 3 months and one year old. The histological score progressed significantly in Nrf2−/− mice at the age of 9 months onward whereas no significant difference between Nrf2−/− and Nrf2+/+ mice was found at 3 months. Recently, reduced levels of Nrf2 have been reported to accelerate age-related senescence in neurological conditions by suppressing the Nrf2/ARE pathway, activating oxidative stress and neuroinflammation^31^. A similar increase in oxidative stress could be responsible for the accelerated disc degeneration found in relation to a lower Nrf2 level.

In this study, we also discovered that induced IVD degeneration became more severe in Nrf2 deficient mice than in WT mice, and although the expression of autophagic-related genes, in particular, Atg5 and Atg7, were significantly upregulated in WT mice with induced IVD degeneration there was no significant difference in the levels of these genes in Nrf2-KO mice. In contrast, HO1 and ULK1 were significantly downregulated in the Nrf2-KO mice. HO1 has been found to modulate degeneration of the induced IVD degeneration in Bach 1 deficient mice by inhibiting oxidative stress^29^. In a recent study, on the effects of sesquiterpene aminoquinones against H_2_O_2_-induced oxidative injury in a human keratinocyte cell line, elevated HO1 expression was associated with increased ARE activity, which, in turn, induced Nrf2 expression^32^. As in our study, Liu et al.^32^ found that the suppression of Nrf2 abolished HO1 expression. Therefore, the suppression of HO1 expression by Nrf2 knockout may result in increased damage from oxidative stress^32^. Moreover, mice deficient in Nrf2 appear to have reduced autophagy as the expression of Atg5 and Atg7 remained unaltered whereas it was significantly increased in Nrf2+/+ mice.

We further analyzed the role of oxidative stress and autophagy by inducing H_2_O_2_ oxidative stress in WT and Nrf2-KO NP cells with and without an autophagy inhibitor. There were increased levels of LC3 puncta under oxidative stress in WT NP cells compared with Nrf2-KO NP cells. TEM and immunofluorescence images indicated the presence of autophagosomes in WT NP cells whereas a decreased number of autophagosomes were observed in Nrf2-KO NP cells. Moreover, the levels of Atg7 were lower in Nrf2-KO NP cells than in WT NP cells, indicating that Nrf2 may be required for H_2_O_2_-induced autophagy in NP cells. Furthermore, Atg7 knockdown blocked the accumulation of LC3-II induced by H_2_O_2_ exposure and inhibited Keap1 degradation. Depletion of Keap1 promotes H_2_O_2_-induced autophagy and increases transcriptional activity of the antioxidant Nrf2. The expression of p62 was significantly increased in Nrf2-KO NP cells compared with Nrf2-WT NP cells. p62 phosphorylation at S349 increased its affinity for Keap1 markedly via its Keap1-interacting region^33,34^. Level of p62(pS349), Atg7 and LC3-II and Keap1 degradation in NP cells treated with H_2_O_2_ and transfected with siAtg7 was analyzed by western blotting. Atg7 knockdown blocked the accumulation of LC3-II and inhibited Keap1 degradation induced by H_2_O_2_ exposure.

Overall, our results indicate that H_2_O_2_-induced oxidative stress was increased in autophagy-deficient cells by an autophagic pathway modulated by Atg7. And a Keap1–Nrf2–p62 feedback loop may contribute to regulate the autophagic pathway.

Other studies attribute the regulation of autophagy under oxidative stress to crosstalk through a feedback loop involving Nrf2^20–22^. Kapuy et al.^20^ claim that the cellular oxidative stress response mechanism is achieved through a negative feedback loop involving Nrf2, mTOR, and AMPK. The mTOR–Nrf2 double negative feedback generates bistability, supporting the separation of autophagy-dependent survival at low stress and cell death at high stress. The authors suggest that an AMPK–mTOR–Nrf2 negative feedback loop provides an oscillatory characteristic of autophagy upon prolonged intermediate levels of oxidative stress, which results in repetitive autophagy stimulation^20^. Hayashi et al.^21^ suggest that autophagy plays a cytoprotective function in oxidative stress-induced necrosis because the decision for cell death in auditory cells under oxidative stress depends on the balance between autophagy and necrosis due to ATP depletion. The authors claim that autophagy was a cell survival mechanism in H_2_O_2_-induced cell death because the suppression of autophagy by Atg7 sensitized the cells whereas activation of autophagy by rapamycin protected the cells^21^. They further proposed that Nrf2 is activated accompanying autophagy and knockdown of Atg7 inhibits Keap1 degrading in a p62-dependent manner to impair autophagy in the feedback of Keap1/Nrf2/p62 pathway. Which supports our theory of Nrf2 in a negative feedback loop in the Keap1–Nrf2–p62 in association with Atg7. Interestingly, a study by Wang et al.^22^ proposed that ESI, a drug isolated from *Elephantopus scaber* L, could significantly induce autophagy in lung cancer cells^22^. ESI was found to increase the expression levels of autophagy markers in a dose-dependent manner by inducing the nuclear translocation of Nrf2 to activate downstream ARE genes, including HO-1 and p62. The authors believe this occurred because ESI-induced p62 could competitively bind with Keap1 and thereby release Nrf2 to activate downstream targets. It would be interesting to determine the effects of ESI in IVD degeneration.

### Conclusion

To conclude, we have found that expression of Nrf2 is decreased in human NP cells and that a deficiency in Nrf2 leads to IVD degeneration during aging. Nrf2 knockout also aggravates IVD degeneration and reduces autophagy gene expression in a mouse model of IVD degeneration. The detrimental effects of oxidative stress were increased in autophagy-deficient cells. Taken together, our results suggest that excessive oxidative stress causes the upregulation of autophagy, and autophagy acts as an antioxidant response activated by a Keap1–Nrf2–p62 feedback loop in IVD degeneration.

## Methods

### Clinical study population/human specimens

Informed consent was given by the patients or relatives to obtain human intervertebral tissue at surgery. The experimental methods were carried out in accordance with approved guidelines and the study was authorized by the ethics committee of the Second Military Medical University. The NP samples with different degrees of IVD degeneration (*n* = 60; age 20 to 79 years, mean age 45.4 years) were obtained from patients who underwent disc resection surgery or spinal fusion to relieve lower back pain. MRI was performed on patients prior to surgery. The assessment of IVD degeneration was performed according to the classification system described by Pfirrmann et al.^27^. The samples were divided into four groups (15 samples per group): non-degenerated (grade I/II), mildly degenerated (grade III), moderately degenerated (grade IV), and severely degenerated (grade V).

### Immunohistochemistry

Immunohistochemistry to assess Nrf2 expression was performed on cryosections of degenerated IVD tissue. The cryosections were incubated in 0.8% hyaluronidase at 37 °C for 60 min, washed with PBS, and then blocked in 0.5% goat serum (40 min, room temperature). Samples were then incubated overnight with polyclonal anti-Nrf2 (1:100), or control rabbit IgG (2 μg/mL) at 4 °C. After washing in PBS, samples were incubated with secondary antibody conjugated to horseradish peroxidase (1:3000, 30 min, room temperature). Washed sections were then incubated with diaminobenzidine (DAB; Solarbio, DA1010), followed by counterstaining with hematoxylin. Images were captured under a microscope.

### Real-time PCR

RNA was extracted from human NP samples using Trizol (Invitrogen, Carlsbad, CA, USA) according to the manufacturer’s instructions and total RNA was measured spectrophotometrically at 260 nm. First strand cDNA synthesis was performed with 500 ng of total RNA in a 10 μL final volume containing 2 μL PrimerScript RT Master Mix (Takara Bio Inc., Shiga, Japan) and 8 μL of RNase-Free dH_2_O. Reverse transcription was performed following manufacturer’s instructions. Real-time PCR was performed using SYBR premix Ex Taq (Takara) with a Step One Plus real-time PCR system (Applied Biosystems, Foster City, CA, USA), according to the manufacturer’s instructions. GAPDH was used to normalize gene expression and the relative amount of each transcript was calculated using the Ct method.

### Experimental animals

The experimental animals: Nrf2−/− C57BL/6J mice and wild-type C57BL/6J mice, were purchased from the Nanjing Biomedical Research Institute of Nanjing University (Nanjing, China). A total of 40 Nrf2−/− mice and 80 wild-type mice were used in the related IVD degeneration model. In total, 40 wild-type mice and 30 Nrf2−/−mice were 3 months old, 10 wild-type mice were 6 months old, 20 wild-type mice and 10 Nrf2−/− mice were 9 months old, and 10 wild-type mice were 1 year old. The ethics committee which reviewed animal experiments at the Second Military Medical University approved this study.

### Surgical induced and aging-related IVD degeneration model

To assess age-related IVD degeneration in vivo, ten mice from each age group (3 months, 6 months, 9 months, and 1 year old) were used. One disc from each mouse was used for analysis. Mice were then euthanized and the C9–C10 caudal discs were harvested. Five specimens per group were stored at −80 °C for mRNA extraction for RT-PCR and for protein extraction for western blotting. The remaining IVD tissues were fixed in 4% paraformaldehyde in PBS (pH 7.4), decalcified in EDTA, paraffin-embedded, and sectioned (5 μm). Sections were stained with hematoxylin and eosin for cellular constituents and Safranin O for proteoglycans.

The annulus needle puncture model to induce IVD degeneration was performed on 10 mice aged 3 months. Nrf2−/− and WT mice were anesthetized with 2,2,2-tribromoethanol (125 mg/kg), then the needle puncture was performed at C9–C10 caudal discs using a 29-gauge needle. Disc height was measured by radiographic analysis at 8 weeks post-puncture to evaluate disc degeneration. The mice were then euthanized and the C9–C10 caudal discs were harvested. Five specimens per group were stored at −80 °C for mRNA extraction for RT-PCR and for protein extraction for western blotting. The remaining tissues were fixed, decalcified, paraffin-embedded, and sectioned. The sections were stained with hematoxylin and eosin and Safranin O. Disc degeneration was then scored using the method described below.

### Scoring system of IVD degeneration

We used the IVD degenerative histological score described in Boos et al.^28^ to evaluate whole IVD in wild-type and Nrf2−/− mice^28^. This grading scheme for the evaluation of IVDs includes a range from 0 to 22 points, where 0 represents no degeneration and 22 represents severe degeneration, and a range for the endplate from 0 to 18 points, where 0 represents no degeneration and 18 represents severe degeneration. The grading system allows the broad evaluation of the whole IVD, including the AF and NP regions. Scoring was assessed by two independent observers.

### NP cells isolation and culture

To isolate NP cells from lumbar discs, animals were euthanized and lumbar IVDs were collected from resected spinal columns. The gelatinous NP was separated from the AF under a dissection microscope and treated with 0.1% collagenase for 3 h. Partially digested NP tissue was maintained in DMEM/F12 and 10% fetal bovine serum supplemented with antibiotics in a humidified atmosphere (5% CO_2_, 37 °C). After a week, NP cells migrated from the partially digested tissue and were allowed to grow to confluence. Primary-passage cells were harvested using 0.25% trypsin-EDTA (1 mM) solution and seeded into culture plates. Second-passage cells were used for the experiments.

### NP cell treatment and siRNA transfection

When NP cells reached confluence, they were trypsinized with 0.25% trypsin/ethylene diamine tetraacetic acid (Gibco, Gaithersburg, MD, USA) and seeded into six 24-well plates at 150,000/10,000 cells/well in the same medium. When cells in the plates reached 90% confluence, we incubated the cells with 400 μM H_2_O_2_ for different times. Pre-designed siRNA and negative control siRNA to silence the mouse Atg7 or Keap1 gene were purchased from Ambion (Cell Signaling Technology, MA, USA). For transfection, before H_2_O_2_ treatment, cells were seeded into 6-well plates, incubated for 24 h, then transfected with Atg7 siRNA (100 nM), Keap1 siRNA (100 nM) or negative control siRNA, using Lipofectamine RNAiMAX Transfection Reagent (Invitrogen) according to the manufacturer’s instructions. After subsequent treatments, cells were harvested for analysis.

### Assessment of the level of ROS

The generation of ROS was detected by a method described in a previous report (10.1038/srep36396). Cells were stained with 2′,7′-Dichlorodihydrofluorescein diacetate (DCFH-DA, Sigma-Aldrich) for 30 min at 37 °C. A microplate reader was used to measure the oxidation of DCFH-DA into dichlorofluorescein (DCF) (excitation 485 nm/emission 520 nm). The results were normalized to the total protein concentration, and ROS levels were presented as a percentage of the control group.

### Transmission electron microscopy

NP cells were trypsinized and fixed with 2.5% glutaraldehyde and 2.0% paraformaldehyde in 0.1 M sodium cacodylate buffer, pH7.4, overnight at 4 °C. After washing in buffer, the samples were fixed in 2.0% osmium tetroxide (1 h, room temperature) and then washed in buffer followed by dH_2_O. The cell pellet was dehydrated through a graded ethanol series, and then infiltrated and embedded in EMbed-812 (Electron Microscopy Sciences, 14900). Sections were stained with lead citrate and observed under a JEOL 1010 electron microscope. Images were captured with a digital camera (Hamamatsu C4742-95) and dedicated software.

### Acridine orange staining

Acridine orange (1 μg/mL; Sigma-Aldrich, St Louis, MI, USA) was added to NP cells and then incubated in the dark. After incubation, media was replaced with PBS and the fluorescence intensity was measured at 488–525 nm for DNA bound green signals or at 488–650 nm for acidic red signal using a microplate reader (Infinite M1000 Pro, Tecan, Switzerland). Acridine orange staining was quantified by normalizing fluorescence readings at 650 nm with those at 525 nm.

### LysoTracker Red staining

NP cells were cultured with H_2_O_2_ or in normal conditions for 24 h. LysoTracker Red DND-99 (50 nM; Thermo Fisher Scientific) was added and cells were incubated in the dark for 30 min at 37 °C. After cells were washed with PBS, they were fixed with 4% paraformaldehyde (Sigma-Aldrich) for 15 min at room temperature in the dark. Cells were then washed with PBS and mounted onto slides with ProLong Gold Antifade Mountant with DAPI (Thermo Fisher Scientific, Waltham, MA, USA) for observation under a microscope. Images of cells were taken from three independent experiments using a Zeiss Axio Imager microscope. Red puncta were quantified by measuring the number in a specific area.

### Immunofluorescence

For the immunofluorescence staining of Nrf2 and LC3 in cultured cells, NP cells were plated on glass coverslips. Then fixed and permeabilized with cold methanol at −20 °C for 15 min, washed with PBS and then blocked with 5% normal goat serum in PBS with 0.3% Triton X-100 for 1 h at room temperature. Cells on coverslips were then incubated with anti-Nrf2 or anti-LC3 antibodies (Cell Signaling Technology) in blocking buffer at a dilution of 1:100 at 4 °C overnight, washed with PBS and then incubated with Alexa Flour-488 Mounted with ProLong Gold Antifade Mountant with DAPI. All mounted slides were visualized using a Zeiss LSM510 confocal microscope.

### Protein extraction and western blot analysis

NP cells, at 70–80% confluence, were washed twice with PBS then resuspended and incubated for 10 min on ice in 500 μl extraction buffer (10 mM HEPES-KCl (pH 7.6), 10 mM KCl, 5 mM MgCl_2_). A further 500 μl of extraction buffer was added with 1% Triton-100 to solubilize plasma membrane and leave the nuclear membrane intact. The supernatant was incubated on ice for 20 min, and 500 μl nuclear isolation buffer (10 mM HEPES-KCl (pH 7.6), 10 mM KCl, 5 mM MgCl_2_) was added. Homogenates were then centrifuged at 600 × *g* (10 min, 4 °C) for separation into the supernatant cytosolic fraction and pellet nuclear fraction. Proteins were extracted using RIPA lysis buffer (Cell Signaling Technology) and protein concentration was determined with a BCA kit (Thermo Fisher Scientific, Shanghai, China).

For western blotting, after electrophoresis on 10 or 12% SDS-PAGE, the samples were transferred to a PVDF membrane (Millipore, Bedford, MA, USA). The membrane was blocked for 1 h with 5% nonfat milk and then incubated with primary antibody for 1 h at room temperature. After washing, the membrane was then incubated with HRP-conjugated goat anti-mouse/rabbit secondary antibodies (1:4000; Proteintech, Chicago, IL, USA) at room temperature for 1 h. The membranes were then visualized using ECL (Bio-Rad, Hercules, CA, USA) and exposed to autoradiographic film.

### Statistical analysis

All measurements were performed at least in triplicate. Data are presented as the mean ± SD. Differences between groups were assessed by the Student’s *t* test and ANOVA with appropriate post hoc analysis. A value of *p* < 0.05 was considered statistically significant.
